# Dietary Effects of Some Plant Extracts on Laying Performance, Egg Quality, and Some Blood Parameters in Laying Hens at Different Cage Densities †

**DOI:** 10.3390/ani13243866

**Published:** 2023-12-15

**Authors:** Nurinisa Esenbuga, Ozlem Ekinci

**Affiliations:** 1 Department of Animal Science, Faculty of Agriculture, University of Ataturk, Erzurum 25240, Türkiye; 2 Department of Poultry, General Directorate of Livestock, Ministry of Agriculture and Forestry, Ankara 06170, Türkiye

**Keywords:** cage density, thyme extract, anise extract, black cumin seed extract

## Abstract

**Simple Summary:**

The cage systems used in commercial laying hen breeding are preferred due to their ease of maintenance, management, and economic advantages. However, a high number of animals per unit area increases the risk of stress and disease. Due to the ban on the use of antibiotics in animal nutrition, plant extracts have taken an important place in the search for alternative additives. Therefore, in our study, the effects of three different plant extracts added to the diets of laying hens raised in different cage densities were examined. As a result, an increased cage density was associated with stress and depression in the feed consumption and metabolic profiles. Supplemental plant extracts improved some laying performance and metabolic profiles.

**Abstract:**

This study was carried out to determine the effects of cage density and anise extract (AE), thyme extract (TE), and black cumin extract (BCE) supplementation in the diet of laying hens on laying performance, egg quality, and some blood parameters. A total of 288 Lohman White commercial hens were blocked according to the location of their cages. The four dietary treatments included a control, basal diet + 250 mg/kg of AE, basal diet + 250 mg/kg of BCE, and basal diet + 250 mg/kg of TE for 12 weeks. The cage density affected egg production (*p* < 0.05), feed consumption (*p* < 0.01), and cracked eggs (*p* < 0.05). Increasing the cage density caused a linear decrease in egg production and feed consumption. Compared to the control, there was a decrease in feed consumption (*p* < 0.01) in the plant extract groups, and in parallel, egg production decreased. An increased cage density did not affect the egg quality traits except the shell strength. The shell strength, yolk color, yolk index, albumen index, and Haught unit were significantly affected by the plant extracts. The cage density and plant extracts had a significant effect on the serum corticosterone and glucose (*p* < 0.01). The highest values of corticosterone and glucose were recorded for dietary TE with a cage density of 4 birds/cm^2^. On the other hand, the lowest values of these parameters were recorded for AE addition with a cage density of 3 birds/cm^2^. As a result, an increased cage density was associated with stress and depression in the feed consumption and metabolic profiles. Supplemental AE, BCE, and TE improved the laying performance and metabolic profiles.

## 1. Introduction

The increase in the need for food in parallel with the increasing population of the world makes efforts to achieve higher and higher quality yields from existing resources more important. In particular, the increase in urbanization and the differentiation of living habits in economic and social terms have made poultry farming a sector. Eggs, which are high in protein, rich in vitamins and minerals, and low in calories, are one of the most important foods. The cage systems used in commercial laying hen breeding are preferred due to their economic advantages, such as facilitating maintenance and management, using automatic systems, housing more animals per unit area, and enabling more egg production [1,2,3]. However, especially due to increasing social concerns regarding animal welfare, it is important to create more suitable conditions when raising laying hens in cages. Nevertheless, in many countries, commercial concerns may prevent this situation and the situation arises that more chickens can be raised per unit area [1]. Nonetheless, the high cage density causes an increase in environmental temperature, a decrease in airflow, and therefore, a rise in body temperature, poor ventilation, an increase in ammonia, and a decrease in performance by preventing access to feed and water. It is stated that there should be a minimum area of 550 cm^2^ per hen in the cage for world-class laying hens [4]. The high number of animals per unit area increases the risk of stress and disease. Stress, on the other hand, leads to the disruption of the intestinal microflora balance and weakening of the immune system. This situation reduces the quantity and quality of the products obtained, leading to a decrease in profitability. Many studies have identified negative effects of stock density on egg production, feed consumption, egg weight, egg quality, body weight, and mortality [5,6]. Feeding strategies are very important in preventing losses in yield and product quality as a result of stress caused by high cage density. Due to the prohibition of the use of antibiotics in animal nutrition, plant extracts have had an important place in the search for alternative additives. For this purpose, supplementation with plant extracts is effective in reducing the stress caused by a high cage density. In recent years, the use of medicinal plants has increased due to their beneficial effects on poultry. In addition, these additives have attracted attention in the poultry industry for their beneficial effects on laying performance and hen health [7,8,9].

Plant extracts are natural feed additives for poultry, which have sweetening, digestive, productivity-enhancing, antibacterial, and anticoccidial properties, and are formed by the combination of ground forms of plants or extracts and oils [10,11,12]. The region where herbal extracts are mainly effective in animals is the digestive system of the animal. They show this effect either by increasing the concentration of the microbial population in the digestive system or by destroying the pathogenic microflora in the digestive tract. This leads to better digestion and absorption of nutrients [11,12].

The most researched aromatic herb is thyme. Thyme (*Thymus vulgaris*) belongs to the Lamiaceae family. The common feature of thyme species that are widely used and traded in Turkey is that they contain essential oils (78–82%), and the main components of these essential oils are thymol (40%) and carvacrol (15%) [13]. These substances are phenolic compounds that give thyme its unique smell and antioxidant properties. A wide variety of pharmacological activities of thymol have been identified, such as antioxidant, antispasmodic, antimicrobial, antiviral, anticancer, anti-inflammatory, and growth enhancer [14,15].

One of the alternatives used as feed additives is black cumin. Black cumin (*Nigella sativa* L.) is also known as black seed and grows in Asian and Mediterranean countries [16]. It is an herbaceous plant that belongs to the Ranunculaceae family and grows especially in Asian and Mediterranean countries. Black cumin contains 35–40% oil, essential oil, saponin, tannin, nigelon, and thymochinon. Black cumin seeds are used in medicine as antispasmodic, anthelmintic, antiseptic, antiarthritic, nerve stimulant, digestive, appetite stimulant, diuretic, antihypertensive, and anticarcinogenic, etc. agents used for such purposes. It also has antioxidant and antibacterial properties due to its ingredients, such as thymoquinone and carvacrol [17].

Anise (*Pimpinella anisum* L.) is an annual aromatic herb belonging to the Apiaceae family. It is mainly cultivated in Southern Europe and Southeast Asia. Anise berries or so-called seeds are the parts of the plant that are used [12]. Anise fruit contains 2–6% essential oil. The most important ingredient of the essential oil is trans-anethole (a powerful phytoestrogen) (80–95%). The seeds of the plant, which carry 1–6% of an ethereal oil called anethole, are widely used in the beverage industry, especially in raki production, to add aroma and fragrance. In this respect, another common use of anise, which has the characteristics of an industrial plant, is for medical (especially alternative medicine) purposes. Anise has been used for its antimicrobial, antioxidant, antibacterial, and antifungal properties over the years. However, limited research has been performed on anise essential oils or extracts [12].

There are studies on the effects of these three plant extracts on the performance of laying hens. However, studies on stress metabolism are insufficient. In this study, we tried to address the question of what extent the stress that occurs with an increasing cage density can be reduced with plant extracts. The aim of the study was to investigate the effects of cage density and anise extract (AE), thyme extract (TE), and black cumin extract (BCE) supplementation to the diet of laying hens on laying performance, egg quality, and some blood parameters.

## 2. Materials and Methods

### 2.1. Animals, Treatments, and Feed

All the experimental protocols adhered to and were approved by the guidelines of the Animal Ethics Committee of Ataturk University (Approval date: 25 December 2009; Decision No.: 09/123). This study was carried out to determine the effects of cage density and some plant extracts supplementation in the basal diet of Lohmann LS White commercial laying hens reared in the poultry houses of the Food and Livestock Application and Research Center of Ataturk University. Approximately 5000 laying hens (Lohmann LS White) from the same parent flock are housed in three aviaries (three tiers) within the same commercial farm. The farm building is one story in height, and production is carried out for experimental purposes. The normal stocking density applied in the poultry houses is 4 cm^2^/hen. All the hens were individually weighed before being placed into their respective cages and randomly assigned to treatment groups. Their average weight (1608.20 ± 25.32) did not differ (*p* > 0.100) between the groups. A total of 288 Lohmann White commercial laying hens, which are 36 weeks old, were blocked according to the location of their cages (48 × 45 × 45 cm, width × depth × height). It is stated that there should be a minimum area of 550 cm^2^ per hen in the cage for world-class laying hens [4]. This experiment was performed with 3 levels of density (3, 4, and 5 hens per cage) and 4 different diets. After two weeks of the adaptation period, laying hens were assigned randomly to three cage densities (3 low-density, 4 normal-density, and 5 high-density hens per cage, providing 720, 540, and 432 cm^2^/hen), each having 6 replicate cages as subgroups [18]. The four dietary treatments included a control (basal diet), basal diet + 250 mg/kg of AE, basal diet + 250 mg/kg of BCE, and basal diet + 250 mg/kg of TE for 12 weeks. The levels of plant extracts were chosen based on previous results reported by [9,12,16,19,20,21]. The experimental diets were formulated to be isocaloric and isonitrogenous and to meet the NRC nutrient requirements. Feed samples were analyzed for dry matter, crude protein, ether extract, crude fiber, and ash contents according to procedures outlined by the Association of Official Analytical Chemists [22]. The metabolizable energy contents of the experimental diets (Table 1) were calculated according to [23]. During the experiment, 16 h lighting was applied, and feed and water were given ad libitum. The basal diet (granule) was obtained from a commercial feed mill in Erzurum (Table 1).

Thyme (*Thymus vulgaris*) belongs to the Lamiaceae family, native to Southern Europe, and popularly known as thyme. Its use as a condiment is disseminated globally [13]. Black cumin (*Nigella sativa* L.) is also known as black seed. It is an herbaceous plant that belongs to the Ranunculaceae family and grows especially in Asian and Mediterranean countries [16]. Anise (*Pimpinella anisum* L.) is an annual aromatic herb belonging to the Apiaceae family. It is mainly cultivated in Southern Europe and Southeast Asia [12]. Plant extracts (liquid form) were obtained from Ege Lokman Co, Manisa, Turkey. According to the information received from the manufacturer, the anise extract (AE) contained 0.83% α-thujone, 0.80% menthone, 2.50% estragole, 12.41% pulegone, 78.44% trans-anethole, and 2.22% carvacrol; the black cumin seed extract (BCE) contained 41% thymocinene, 11% p-cymene, 8.5% carvacrol, 4.7% terpineol, 2% t-anethole, and 4.4% linalool; and the thyme extract (TE) contained 0.86% L-linalool, 1.74% borneol, 9.58% thymol, and 87.81% carvacrol.

### 2.2. Samples and Data Collection

The egg production, cracked eggs, and feed consumption were measured daily; the egg weight was measured bi-weekly; and the body weights were measured at the beginning and the end of the experiment. The feed conversion ratio (FCR) was expressed as a kilogram of feed consumed per kilogram of egg produced.

Eighteen eggs were randomly collected from each group at the beginning, middle, and end of the experiment and stored for 24 h at room temperature before the determination of egg quality. They were calculated using the following equations: Shell strength (kg/cm^2^) was determined by using a machine with the spiral pressure system (Yasef Kuran 01, Samsun, Turkey); shape index (%) = (egg width, cm/egg length, cm) × 100; shell thickness (mm × 10^−2^) was determined in 3 different parts (upper and lower ends and middle) by using a micrometer; yolk color was determined by using commercially available yolk color fan according to the CIE standard colorimetric system (Yolk Colour Fan, the CIE standard colorimetric system, F. Hoffman-La Roche Ltd., Basel, Switzerland); yolk index (%) = (yolk height, mm/yolk diameter, mm) × 100; albumen index (%) = (albumen height, mm/average of albumen length, mm and albumen width, mm) × 100, and Haugh unit = 100 × log(AH + 7.57 − 1.7 × EW^0.37^), where AH = albumen height, mm and EW = egg weight, g. The albumen and yolk height were measured with a micrometer. The egg yolk diameter, albumen width and albumen length (mm) were measured with a digital caliper.

At the middle and the end of the experimental period, 9 animals from each group were selected and 5 mL blood samples were taken from the wing vein using heparinized tubes after 12 h of fasting. The serum was centrifuged at +4 °C, 3000× *g* for 5 min. Aliquots were kept at −20 °C until the laboratory analyses. The glucose was determined by using commercial kits (DDS Spectrophotometric Kits, Istanbul, Turkey), and the corticosterone concentrations were determined using commercially available radioimmune assay sets (Byk-Sangtec Diagnostica, Dietzenbach-Germany; Immulite 2000, DPC, Los Angeles, CA, USA).

### 2.3. Statistical Analysis

The factorial arrangements of the three cage densities, four plant extracts, and time were tested in a complete randomized block design experiment. A two-way ANOVA was conducted using the GLM procedure (SPSS, 20.0). Data on the laying parameters, egg quality, and blood parameters were analyzed using the SPSS statistical package. The GLM procedure included the main effects of the cage density, plant extract, time, and 2- and 3-way interactions between these factors. When a significant interaction between the main effects was observed, Duncan’s test was used to compare the differences among the groups. Polynomial contrasts were created to determine the effect of the cage densities.

## 3. Results

### 3.1. Laying Performance

**Figure 1:**
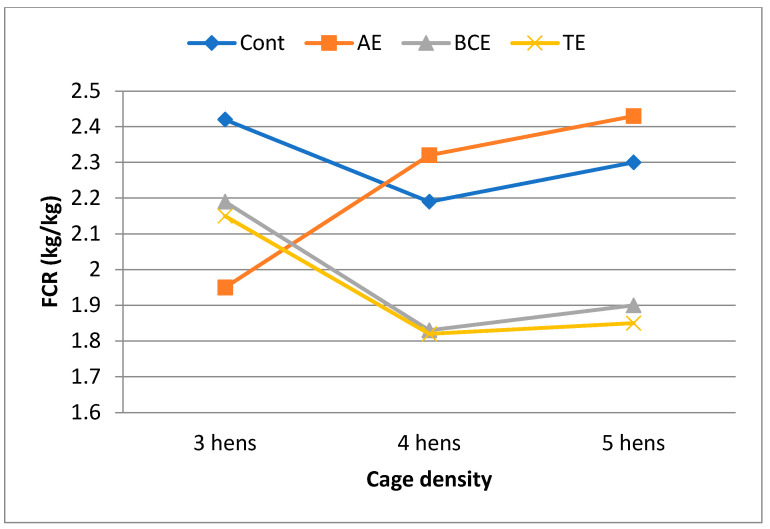
The effect of cage density by plant extract interaction on the food conversion ratio (FCR) (SEM = 0.08 and *p* < 0.05). (Cont: Control, AE: Anise extract, BCE: Black cumin seed extract, TE: Thyme extract).

**Figure 2:**
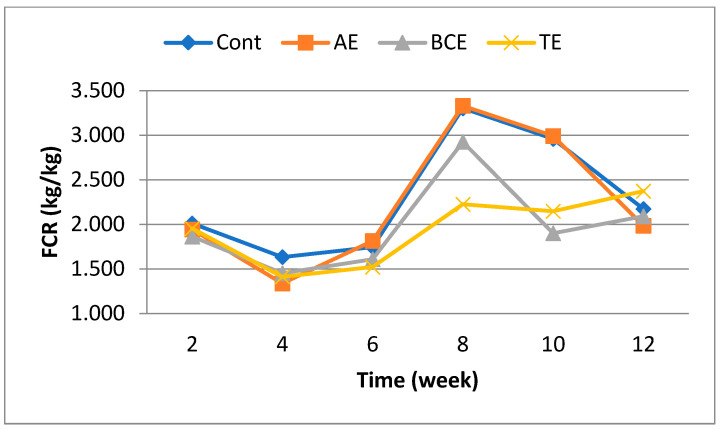
The effect of plant extracts by time interaction on the food conversion ratio (FCR) (SEM = 0.07 and *p* < 0.01). (Cont: Control, AE: Anise extract, BCE: Black cumin seed extract, TE: Thyme extract).

Table 2 presents the effect of the cage density and plant extracts on laying performance. The cage density affected egg production (*p* < 0.05), feed consumption (*p* < 0.01), and cracked eggs (*p* < 0.05). Hens placed in high-density cages consumed a less amount of feed and produced a less amount of eggs than hens placed in low- and normal-density cages. Also, hens in low-density cages tended to produce more cracked eggs than hens in high-density cages (0.786, 0.279, and 0.238%). The effect of the plant extracts on egg production, feed consumption, and FCR was significant, and they had negative effects on egg production and feed consumption compared with the control (*p* < 0.01). The plant extracts did not alter egg weight and cracked eggs. There were variations in egg production, feed consumption, egg weight, FCR, and cracked eggs (*p* < 0.0001) as the experiment continued. The initial body weight, final body weight, and body weight changes in all the groups were not affected by the cage density and AE, BCE, and TE supplementation. There were cage density by plant extract interaction effects on FCR (*p* < 0.043), cage density by time interaction on feed consumption (*p* < 0.0001), and plant extract by time interaction effects on egg weight (*p* < 0.015) and FCR (*p* < 0.005). However, egg production, feed consumption, egg weight, and cracked eggs were not significantly affected by the interaction between cage density and plant extract addition. While the AE group had the lowest FCR value in low-density cages, the highest FCR values were obtained for the AE group in normal- and high-density cages (Figure 1). The FCR values of the BCE and TE groups decreased in parallel with the increase in cage density. The FCR was low until the sixth week of the experiment, then it increased in the eighth week, and at the end of the experiment, similar values were obtained in the first week (Figure 2). Concerning the interaction between cage density and plant extract supplementation, it could be observed that the lowest FCR was achieved in the BCE and TE groups at a cage density of 4 birds/cm^2^ (Figure 1). There were variations in egg production, feed consumption, egg weight, FCR, and cracked egg (*p* < 0.0001) as the experiment continued.

### 3.2. Egg Quality

The effect of cage density and plant extracts on some egg quality traits is shown in Table 3. An increased cage density did not affect the egg quality traits except the shell strength. The shell strength was the lowest in the low-density cages. As the cage density increased, the shell strength improved linearly.

The effects of AE, BCE, and TE supplementation on the egg quality parameters were variable (Table 3). It was determined that plant extract supplementation had no significant effect on the shape index, shell thickness, and shell weight. The shell strength, yolk color, albumen index, yolk index, and Haught unit were significantly affected by the supplementation of the diet with AE, BCE, and TE. While the albumen index and Haugh unit decreased notably in the chickens supplemented with plant extracts, the shell strength and yolk color increased. The plant extract groups had the highest shell strength. Although AE, BCE, and TE added to the diets of laying hens caused an increase in the yolk color compared to the control, they caused a decrease in the Haugh unit. The addition of AE to the diet decreased the yolk index when compared to the control, TE, and BCE groups. The highest albumen index values were obtained for the control and BCE groups, while the AE and TE groups had the lowest albumen index values. There was no cage density by plant extract interaction effect on the egg quality parameters. When the plant extract by the time interaction is examined, it is seen that the shell thickness and yolk index values are affected.

### 3.3. Blood Parameters

Table 4 summarizes the effects of cage density and AE, BCE, and TE supplementation on corticosterone and glucose. The results in Table 4 show that the cage density and inclusion of plant extracts in the hens’ diet had a significant effect on the serum corticosterone (*p* < 0.043) and glucose (*p* < 0.0001). The low-density cages showed significantly lower serum corticosterone (3.72, 3.91, and 3.95 ng/mL, respectively) and glucose (276.87, 282.18, and 290.77 mg/mL, respectively) than the normal-density and high-density cages.

AE, BCE, and TE added to the diet significantly affected the serum glucose and corticosterone levels. The lowest serum glucose and corticosterone levels were obtained for the AE group, while the highest levels were determined for the TE group. Moreover, there was a cage density by plant extracts interaction effect on the serum corticosterone and glucose concentrations (*p* < 0.0001).

## 4. Discussion

In modern laying hen farming, adequate space availability is highly related to productivity and animal welfare. Insufficient space allocated to chickens prevents the animals from displaying natural behavior, resulting in decreases in productivity [1,18]. In many studies on the effects of cage density on egg-laying performance, the results appear to be inconsistent. This may be due to the difference in the breeds used and nutritional conditions. In this experiment, the hens in high-density cages consumed less feed and consequently produced fewer eggs than those in low- and normal-density cages. Additionally, more cracked eggs were obtained from the low-density cages than from the high-density cages. Numerous studies involving stress associated with an increased cage density (540 vs. 360 hen/cm^2^) have shown negative effects on laying performance [2,3]. In this regard, Sohail et al. [24] found that increasing the cage density (413 or 310 birds/cm^2^) leads to a significant decrease in egg production and feed consumption. Kang et al. [25] reported that hens stocked at a high density (10 birds/m^2^) achieved less in terms of egg production and feed consumption compared to other cage densities (5, 6, and 7 birds/m^2^). Zhang et al. [26] attributed the decrease in performance at a high density (10 birds cage (0.54 m^2^)) to the increase in the ambient temperature and the decrease in the airflow, and consequently, the decrease in body temperature. In a study conducted by Wan et al. [18], the effect of cage density on two different breeds was examined and it was reported that the laying rate of chickens raised in low-density cages was significantly higher than in high-density cages. Although some researchers reported that egg production, feed consumption, and FCR decreased as the cage density increased, some researchers found that egg production was not affected by the cage density [2,3,27]. The researchers stated that the decrease in egg production, feed consumption, and FCR may be due to the decrease in the feeder space for the chickens and disturbed social behaviors with the increase in the cage density [2,3]. In this study, hens housed at three birds per cage produced significantly higher cracked egg percentages than those at four and five birds per cage. The reason for the higher percentage of cracked eggs in the low-density chickens is not fully explained but may be the result of increased bird activity. Contrary to the results of this study, Sarica et al. [28] (500, 666.7, 1000, and 2000 birds/cm^2^) and Kahraman et al. [29] (495 and 412 birds/cm^2^) did not report any cracked egg ratio difference cage-density groups.

Similar to this study, Hayırlı et al. [3] reported that the effect of cage density on body weight was insignificant. However, some researchers reported that the body weight decreased as the cage density increased [30,31,32].

Actually, the mechanisms of plant additives are not very clearly defined yet. Dhama et al. [7] and Yadav et al. [9] reported that plant extract or oil may improve intestinal barrier integrity in chickens. In addition, these positive results could be, at least in part, due to the herbal products’ antioxidant and antibacterial effects on the intestine [7,8]. Supplementation with plant extracts reduced egg production and feed consumption. The decrease in feed consumption may be due to plant extracts reducing the pathogenic microflora and thus improving gut ecology and/or increasing the digestibility of nutrients [14,26]. However, it was observed that egg production decreased in the groups that consumed the plant extract compared to the control. This may be due to the decrease in feed consumption. Contrary to this study, Kaya et al. [33], Florou-Paneri et al. [34], Bolukbası et al. [35] and Bayram et al. [19] reported that the addition of AE, BCE, and TE to laying hen diets had no significant effect on feed consumption, egg production, and FCR. Similar to this study, Akhtar et al. [36] showed that the inclusion of BCE (0.0, 0.5, and 1.5%) in the diets of the laying hens improved FCR. Florou-Paner et al. [34], Botsoglou et al. [10] and Bolukbaşı et al. [35] stated that the addition of anise, thymine, and black cumin oil or extract did not have a significant effect on egg weight. These results are similar to the findings of our study, in which the inclusion of plant extracts in the layer diet did not affect the egg weight and cracked egg ratio. Although there was no statistical significance in terms of the egg weights, the relative differences between the groups may have been caused by the effect of the plant extracts. However, Akhtar et al. [36], Yalçın et al. [20], Aydın et al. [37], and Denli et al. [38] reported that anise, thyme and black cumin added to the diet of laying hens increased the egg weight. Data regarding the effects of plant extracts on the body weight of laying hens are controversial. Unlike the results obtained in this study, some researchers stated that anise, black cumin, and thyme oil or extracts added to the diet significantly increased the body weight compared to the control group [21,39,40].

In this study, the egg quality parameters except the shell strength were not affected by the cage density. Similar to this study, some authors reported no changes in the egg quality parameters in response to an increased cage density [2,3,24,25]. The shell strength was the lowest in the low-density cages. As the cage density increased, the shell strength improved linearly (2.17, 2.56, and 2.71 kg/cm^2^). In addition, the shell strength increased in the groups fed with AE, BCE, and TE compared to the control. The reason for the low shell strength of the C and AE groups in the three-cage density group could not be explained. In general, it is reported that the egg quality parameters do not change with dietary manipulations [41]. In contrast to this study, some researchers have stated that the addition of some plant extracts to the layer diet did not have a significant effect on the shell strength [11,42]. In line with our study, Kaya et al. [33], working with different doses of plant extract mixture (PEM), reported that the shell strength increased linearly as the PEM (500, 750, 1000 mg/kg,) increased. Also, Liu et al. [43] reported that there was an increase in the shell strength. This suggests that plant extracts may regulate calcium metabolism by affecting hormones and thereby improving eggshell quality, but the specific mechanism needs to be further investigated. Another study stated that supplementation with anise (0.25%) and thyme (0.25%) did not affect the eggshell thickness or weight in laying hens [44]. Also, Florou-Paner et al. [34] and Yalcin et al. [20] reported that AE, BCE, and TE supplementation did not alter some egg quality parameters. Eggs from hens fed either AE, BCE, and TE presented significantly (*p* < 0.001) different yolk colors compared to the control group. The increase observed in the yolk color may be due to the transition of the pigments in the AE, BCE, and TE extracts added to the diet into the egg yolk. Contrary to this study, Kaya et al. [33] observed that the yolk color, albumen index, yolk index, and Haugh unit were not affected by the addition of plant extract mixture (including thyme oil, origanum oil, garlic oil, anise oil, and fennel oil) to layer diets. There is little published information on the effect of dietary plant extracts on the egg yolk color, yolk index, albumin index, and Haugh unit. In addition, there are no studies on the effect of diet anise on the egg yolk color of laying hens.

The stress process has three stages. In chickens, by stimulating the central nervous system of the stress factor, catecholamines (Nor-epinephrine and epinephrine) are secreted from the adrenal medulla, and in this way, ready-to-use energy-source glycogen is converted into glucose via glycogenolysis. Glucose is the most important monosaccharide that plays a role in energy metabolism. Corticosterone is the major steroid hormone released by the avian adrenal gland, and it can be used as a stress marker to assess the welfare of chickens [2,3,18,45]. Along with the secretion of corticosterone, body reserves (protein, fat, etc.) are activated for glucose synthesis via gluconeogenesis. The secretion of the corticosterone hormone in animals under stress continues until the stress factor is eliminated or corticosterone is depleted in the adrenal cortex. Depending on the level of stocking density, corticoid concentrations and blood glucose increase as well as humoral and cell-mediated immunity being impaired [45,46].

In this study, the corticosterone and glucose concentrations in the low-density cages were significantly lower than those in the medium- and high-density cages. This means that a high cage density can cause stress in chickens. Similar to the results of this study, Hayırlı et al. [3], Jones et al. [46] and Kaya et al. [47] noted that there was a positive relationship between the corticosterone level and cage density and that increased stress with the cage density increased the plasma corticosterone level. On the other hand, there are studies reporting that the cage density does not affect the immune response and stress parameters in laying hens [31]. The results obtained in the study are similar to the findings of Hayırlı et al. [2] and Kang et al. [25], who reported that the serum glucose levels increase as the number of chickens placed per unit area in laying hens increases. On the other hand, unlike our research findings, Kocaoglu et al. [48] reported that the cage density did not have a significant effect on the serum glucose levels, while Yoruk et al. [41] reported that the serum glucose levels decreased as the cage density increased.

The plant extracts significantly affected corticosterone and glucose metabolism. The highest values of corticosterone and glucose were recorded for dietary TE with a cage density of 4 birds/cm^2^. On the other hand, the lowest values of these parameters were recorded for AE addition with a cage density of 3 birds/cm^2^. Contrary to the results obtained in this study, Zhang et al. [26] reported that plant extracts had no effect on corticosterone and glucose. Kocaoglu et al. [48], after adding L-carnitine to the diet, reported that the increase in the serum glucose level may be due to the positive effect of carnitine on energy metabolism. Gholamrezaie et al. [49] stated that the effects of different natural products on the immune system were complex. It was suggested that antioxidants regulate corticosteroid synthesis in the adrenal glands and significantly improve certain immune responses. It had been reported that it had a direct effect on the stimulation of the lymphatic tissue of the digestive system and an indirect effect on the change in the gastrointestinal microbial population [49]. The differences in the results of this study and other research findings may be due to the genotype, age, and spawning periods of the animals used, or to factors such as the level of plant extracts and the method of obtaining the extracts.

## 5. Conclusions

In this experiment, the effects of cage density and some plant extracts, such as AE, BCE, and TE, on the laying performance, egg quality, and metabolic profile were evaluated. The cage density affected the egg production, feed consumption, and cracked eggs. Hens placed in high-density cages consumed a less amount of feed and produced a less amount of eggs than hens placed in low- and normal-density cages. Compared to the control, there was a decrease in feed consumption in the AE, BCE, and TE groups, and in parallel, egg production and FCR decreased. With an increasing cage density, glucose and corticosterone increased linearly for the hens placed in low-, normal-, and high-cage density. AE, BCE, and TE added to the diet significantly affected the serum glucose and corticosterone levels. The highest values of corticosterone and glucose were recorded for dietary TE with a cage density of 4 birds/cm^2^. On the other hand, the lowest values of these parameters were recorded for AE addition with a cage density of 3 birds/cm^2^. In conclusion, an increased cage density was associated with stress and depression in the feed consumption and metabolic profiles. Supplemental AE, BCE, and TE improved the laying performance and metabolic profiles.

## Figures and Tables

**Table 1:** Ingredients and composition of the basal feed.

Ingredients	(%)	Chemical Analysis (Analyzed on a Dry Matter Basis)	
Corn	58.62	Dry matter (%)	87.9
Soybean meal	27.35	Crude Protein (%)	17.9
Limestone	9.00	Ether extract (%)	4.9
Soybean oil	3.00	Crude fiber (%)	4.49
Dicalcium phosphate	1.15	Crude ash (%)	11.7
Vitamins and mineral premix ^1^	0.2	ME, (kcal/kg^−1^) ^2^	2740
Salt	0.50	Calcium (%)	3.56
Methionine (DL-methionine)	0.13	Phosphorus (%)	0.6
Lysine (L-lysine hydrochloride)	0.05		

^1^ Per kilogram contained: vitamin A 12,000,000 IU; vitamin D3 2,500,000 IU; vitamin E, 30,000 mg; vitamin K 34,000 mg; vitamin B1, 3000 mg; vitamin B2, 6000 mg; nicotinamide, 30,000 mg; Ca-D-pantothenate, 10,000 mg; vitamin B6, 5000 mg; vitamin B12, 15 µg; folic acid, 1000 mg; D-biotin, 50 mg; cholin, 300,000 mg; vitamin C, 50,000 mg; Mn, 80,000 mg; Fe, 60,000 mg; Zn, 60,000 mg; Cu, 5000 mg; I, 2000 mg; Co, 500 mg; Se, 150 mg; ^2^ ME: Metabolizable energy calculated according to TSE (1991).

**Table 2:** The effect of cage density and AE, BCE and TE supplementation on laying performance.

		Egg Production (%)	Feed Consumption (g)	Egg Weight (g)	FCR	Cracked Egg (%)	Initial Body Weight (g)	Final BW (g)	Relative BW Change (%)
Cage density	3	84.52 ^a^	103.15 ^a^	64.96	2.182	0.786 ^a^	1605.8	1623.5	3.46
	4	84.71 ^a^	101.65 ^a^	67.24	2.04	0.279 ^b^	1610.4	1654.9	2.82
	5	82.94 ^b^	98.43 ^b^	66.30	2.12	0.238 ^b^	1635.9	1629.0	−0.35
	SEM	1.07	1.08	2.14	0.07	0.12	16.76	21.37	1.43
Plant extract	Cont	89.29 ^a^	115.56 ^a^	64.29	2.31 ^a^	0.122	1622.40	1675.1	3.33
	AE	81.66 ^b^	95.37 ^b^	63.48	2.23 ^a^	0.523	1595.55	1637.2	2.73
	BCE	83.67 ^b^	97.21 ^b^	66.39	1.97 ^b^	0.423	1585.52	1641.1	3.70
	TE	81.67 ^b^	94.16 ^b^	70.52	1.94 ^b^	0.478	1600.74	1589.6	−0.52
	SEM	1.24	1.25	2.47	0.08	0.14	29.02	48.40	1.66
Time	1	80.63 ^b^	93.70 ^cd^	66.72 ^b^	1.94 ^cd^	0.754 ^a^	-	-	-
	2	87.48 ^a^	96.01 ^cd^	84.18 ^a^	1.46 ^e^	0.800 ^a^	-	-	-
	3	91.66 ^a^	97.61 ^c^	65.16 ^b^	1.67 ^de^	0.727 ^a^	-	-	-
	4	82.49 ^b^	108.55 ^a^	61.76 ^bc^	2.95 ^a^	0.010 ^b^	-	-	-
	5	82.10 ^b^	92.68 ^d^	55.72 ^c^	2.50 ^b^	0.015 ^b^	-	-	-
	6	79.98 ^b^	104.89 ^b^	63.47 ^bc^	2.16 ^c^	0.014 ^b^	-	-	-
	SEM	1.52	1.53	3.02	0.10	0.16	-	-	-
ANOVA	P > F								
Cage Density (CD)		0.049	0.008	0.750	0.376	0.028	0.128	0.543	0.062
Linear effect		0.298	0.002	0.658	0.575	0.015	0.145	0.258	0.873
Quadratic effect		0.454	0.516	0.539	0.070	0.117	0.432	0.659	0.845
Plant Extract (PE)		0.0001	0.0001	0.182	0.002	0.150	0.465	0.119	0.264
Time (T)		0.0001	0.0001	0.0001	0.0001	0.0001	-	-	
CD × PE		0.475	0.659	0.268	0.043	0.216	0.803	0.589	0.907
CD × T		0.306	0.0001	0.985	0.691	0.412	-	-	
PE × T		0.347	0.119	0.015	0.005	0.808	-	-	
CD × PE × T		0.978	0.769	0.755	0.612	0.972	-	-	

^a,b,c,d^ Means within a column with no common superscripts differ significantly (*p* < 0.05). Relative body weight (BW) change = [(Final BW − initial BW)/initial BW] × 100. FCR = Feed conversion ratio (kg feed consumed/per kg egg produced). Cont: Control, AE: Anise extract, BCE: Black cumin seed extract, TE: Thyme extract.

**Table 3:** The effect of cage density and AE, BCE, and TE supplementation on egg quality.

		Shell Strength (kg/cm^2^)	Shape Index (%)	Shell Thickness (mm × 10^−2^)	Shell Weight	Yolk Color	Yolk Index	Albumen Index	Haugh Unit
Cage density	3	2.37 ^c^	76.15	0.42	7.71	12.25	42.37	10.02	88.81
	4	2.56 ^b^	75.44	0.43	7.77	12.39	42.47	9.69	87.71
	5	2.71 ^a^	75.97	0.42	7.90	12.26	42.95	9.88	87.92
	SEM	0.11	0.29	0.01	0.09	0.08	027	0.19	0.69
Plant extract	Cont	2.16 ^b^	76.55	0.42	7.78	11.87 ^b^	42.99 ^a^	10.37 ^a^	90.73 ^a^
	AE	2.54 ^a^	75.32	0.43	7.83	12.30 ^a^	41.66 ^b^	9.64 ^b^	87.34 ^b^
	BCE	2.59 ^a^	76.00	0.42	7.72	12.50 ^a^	42.65 ^a^	9.83 ^ab^	86.97 ^b^
	TE	2.64 ^a^	75.54	0.43	7.85	12.54 ^a^	43.09 ^a^	9.62 ^b^	86.97 ^b^
	SEM	0.13	0.34	0.01	0.11	0.10	0.31	0.21	0.80
Time	1	2.22 ^b^	75.61	0.38 ^b^	7.10 ^b^	12.60	42.70	10.17 ^a^	89.51 ^a^
	2	2.63 ^a^	76.33	0.44 ^a^	8.14 ^a^	12.11	42.90	10.03 ^a^	89.01 ^a^
	3	2.59 ^a^	75.60	0.45 ^a^	8.14 ^a^	12.19	42.19	9.39 ^b^	85.91 ^b^
	SEM	0.11	0.29	0.01	0.09	0.08	0.27	0.19	0.69
ANOVA	P > F								
Cage Density (CD)		0.003	0.208	0.240	0.324	0.436	0.266	0.446	0.494
Linear		0.001	0.663	0.541	0.142	0.907	0.129	0.585	0.366
Quadratic		0.377	0.086	0.115	0.762	0.200	0.561	0.252	0.442
Plant Extract (PE)		0.040	0.058	0.150	0.842	0.0001	0.006	0.047	0.003
Time (T)		0.018	0.131	0.0001	0.0001	0.0001	0.171	0.008	0.0001
CD × PE		0.212	0.123	0.665	0.912	0.993	0.290	0.512	0.675
CD × T		0.449	0.225	0.328	0.244	0.773	0.648	0.451	0.219
PE × T		0.199	0.052	0.010	0.281	0.699	0.010	0.428	0.307
CD × PE × T		0.147	0.723	0.961	0.463	0.516	0.539	0.571	0.341

^a,b,c^ Means within a column with no common superscripts differ significantly (*p* < 0.05). Cont: Control, AE: Anise extract, BCE: Black cumin seed extract, TE: Thyme extract.

**Table 4:** The effect of cage density and AE, BCE, and TE supplementation on corticosterone and glucose.

		Corticosterone (ng/mL)	Glucose (mg/mL)
Cage density	3	3.72 ^b^	276.87 ^c^
	4	3.91 ^a^	282.18 ^b^
	5	3.95 ^a^	290.77 ^a^
	SEM	0.05	1.65
Plant extract	Cont	3.91 ^b^	286.40 ^b^
	AE	3.69 ^c^	265.83 ^d^
	BCE	3.81 ^bc^	278.75 ^c^
	TE	4.13 ^a^	302.12 ^a^
	SEM	0.06	1.90
Time	1	3.57 ^b^	253.40 ^b^
	2	4.19 ^a^	313.14 ^a^
	SEM	0.04	1.34
ANOVA	P > F		
Cage Density (CD)		0.043	0.0001
Linear effect		0.83	0.0001
Quadratic effect		0.528	0.418
Plant Extract (PE)		0.0001	0.0001
Time (T)		0.0001	0.0001
CD × PE		0.0001	0.0001
CD × T		0.934	0.161
PE × T		0.067	0.0001
CD × PE × T		0.137	0.0001

^a,b,c,d^ Means within a column with no common superscripts differ significantly (*p* < 0.0). Cont: Control, AE: Anise extract, BCE: Black cumin seed extract, TE: Thyme extract.

## Data Availability

The original data are available on request from the corresponding author.
